# Sex-Differences in Post-Procedural Pain Experiences After Thermal Liver Ablations for Liver Tumors: A Retrospective Study

**DOI:** 10.1007/s00270-024-03851-5

**Published:** 2024-09-04

**Authors:** R. R. M. M. Knapen, M. C. Homberg, A. J. R. Balthasar, K. Jans, S. M. J. Van Kuijk, S. W. de Boer, E. A. C. Bouman, C. Van der Leij

**Affiliations:** 1 Department of Radiology and Nuclear Medicine, Maastricht University Medical Center+, Maastricht, The Netherlands; 2 School for Cardiovascular Diseases (CARIM), Maastricht University, Maastricht, The Netherlands; 3 Department of Anesthesiology and Pain Medicine, Maastricht University Medical Center+, Maastricht, The Netherlands; 4 Department of Cardiology, VieCuri Medical Center, Venlo, The Netherlands; 5 Department of Epidemiology, Maastricht University Medical Center+, Maastricht, The Netherlands; 6 Research Institute for Oncology and Reproduction, Maastricht University, Maastricht, The Netherlands

**Keywords:** Microwave ablation (MWA), Thermal ablation, Numerical rating scale (NRS), Sex differences, Pain intensity

## Abstract

**Introduction:**

Literature shows differences in pain experiences between sexes. The exact influence of thermal liver ablation on experienced pain is still not well-known. This study aims to investigate the maximum pain intensity at the recovery between men and women after percutaneous thermal liver ablation.

**Methods:**

Patients treated with percutaneous thermal liver ablation (radiofrequency or microwave ablation) in Maastricht University Medical Center + between 2018 and 2022 for primary or secondary liver tumors were included retrospectively. Outcomes included maximum numerical rating scale (NRS, scale:0–10) score at the recovery room, prevalence of post-procedural pain (defined as NRS score ≥ 4), duration of anesthesia, length of stay at recovery, and complications. Regression analyses were adjusted for age, ASA-score, BMI, tumor type, maximum diameter of lesion, chronic pain in patients’ history, and history of psychological disorder.

**Results:**

183 patients were included of which 123 men (67%). Results showed higher average maximum NRS scores in women patients compared to men (mean:3.88 versus 2.73), but not after adjustments (aß:0.75, 95%CI:−0.13–1.64). Women suffered more from acute post-procedural pain (59% versus 35%; aOR:2.50, 95%CI:1.16–5.39), and needed analgesics more often at the recovery room (aOR:2.43, 95%CI:1.07–5.48) compared to men. NRS score at recovery arrival did not significantly differ (aß:0.37, 95%CI:-0.48–1.22). No differences were seen in the length of stay at the recovery, duration of anesthesia, procedure time, and complication rate. Location of the tumor (subcapsular or deep), total tumors per patient, and distinction between primary and secondary tumors had no influence on the NRS.

**Conclusion:**

This retrospective single-center study shows higher post-procedural pain rates after thermal liver ablation in women, resulting in higher analgesics use at the recovery room. The results suggest considering higher dosage of analgesics during thermal liver ablation in women to reduce post-procedural pain.

**Level of Evidence 3:**

Non-controlled retrospective cohort study.

**Graphical Abstract:**

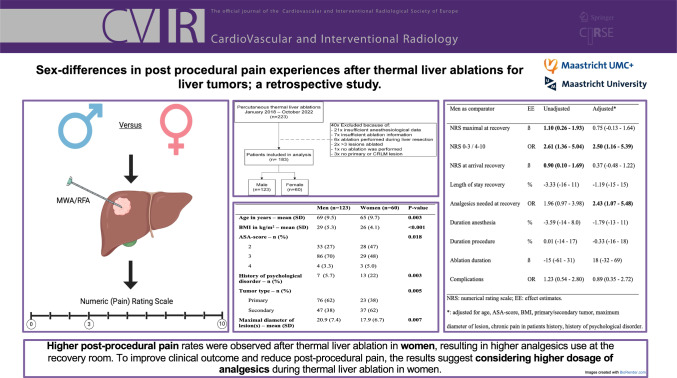

**Supplementary Information:**

The online version contains supplementary material available at 10.1007/s00270-024-03851-5.

## Introduction

Percutaneous radiofrequency (RFA) and microwave frequency (MWA) thermal liver ablation are minimally invasive first-line treatment options for patients with hepatocellular carcinoma (HCC) or colorectal liver metastases (CRLM) [1, 2]. According to the Barcelona clinic liver Cancer (BCLC) prognosis and treatment strategy, very early stage and some early stage HCCs should be treated in first-line with thermal ablation [3]. In patients with CRLM, liver surgery still remains the standard treatment option[4, 5]. However, in these patients thermal liver ablation is used increasingly over the last years with low complication rates [6, 7].

Approximately 10% of the patients treated with thermal ablation develop moderate to severe acute postprocedural pain [8]. After surgery this rate is 30–80%, even after minor procedures [9]. Ineffective post-procedural pain management leads to increased length of hospital stay, increased opioid use, delirium in elderly patients, and thromboembolic complications. Limiting the experienced postprocedural pain can be of great influence on clinical outcomes [10]. Several preoperative predictors are known to be associated with pain control after surgery, such as age, sex, Body Mass Index (BMI), and anxiety [10, 11]. In thermal liver ablation, pre-operative pain predictors are less well-known. Ablation volume, increase aspartate aminotransferase levels, and tumor location, however, seem to increase the risk of post-ablation pain [12, 13].

A better understanding of pain predictors can help interventional oncologists and other clinicians to anticipate to pain stimuli experienced during thermal ablation, in order to decrease post-procedural pain. One of these potential pain predictors is sex. Sex is a well-known pain predictor in surgery, however, is has not been thoroughly investigated as a potential predictor of pain in thermal liver ablation [10, 11]. The study’s aim was to investigate pain intensity after thermal liver ablation (RFA and MWA) between men and women during the period at the recovery.

## Material and Methods

### Design

This retrospective, single-center, cohort study was performed at Maastricht University Medical Center (MUMC +), a tertiary care hospital in the Netherlands. The local medical ethics committee approved the study protocol and the need to obtain informed consent was waived (METC 2022-2222).

Data for this study were collected from the hospital’s electronic health records (SAP GUI 7.60) and the patient data management system (PDMS) and stored in an online secured electronic database (Castor EDC, V2022.5.4.0). Patient’s information was pseudonymized using a unique code. Only the associated researchers had access to the code, which was saved on dedicated hardware. This study was conducted using the STROBE guidelines.

### Participants

Patients treated with percutaneous thermal liver ablation for primary or secondary liver tumors between January 2018 and May 2022 were included. Other inclusion criteria were: ≥ 18 years and ablation performed with CT- or ultrasound guidance. Patients were excluded when anesthesiology or thermal ablation data were insufficient, when four or more liver lesions were treated, or when ablation was performed during (laparoscopic) liver resection at the operation room.

### Thermal Liver Ablation Procedure

All patients received an ultrasound prior to the thermal ablation and were routinely checked by the anesthesiologist. During the thermal liver ablation the duration of ablation, the used wattage, and the used analgesics were determined by the interventional oncologists. All ablation needles were placed ultrasound and/or CT-guided and performed by an experienced interventional radiologist. For ablation, the NeuWave Microwave ablation system (Ethicon; Johnson & Johnson), or the HS Amica generator (HS; Hospital Service) was used.

### Anesthesia Technique

Thermal liver ablation is generally performed under procedural sedation (PSA), which included propofol and an opioid. During PSA remifentanil or alfentanil were administered. General anesthesia is an alternative for patients who do not meet the inclusion criteria for PSA for thermal liver ablation (e.g. BMI > 35, sleep apnea, > 3 lesions, prior unsuccessful PSA). Regardless the anesthesia technique, all patients received lidocaine or bupivacaine as supplementary loco-regional anesthesia before thermal liver ablation started. Piritramide or morphine in combination with paracetamol was administered to prevent postprocedural pain. The addition of metamizole or/ and esketamine was based on the clinician’s experience and preference. Following the ablation, patients were transported to the recovery room to monitor vital and pain parameters. At the recovery room no specific protocol was followed regarding analgesics use.

### Outcome Measures

Clinical outcomes were the maximal numeric ranking scale (NRS) score at the recovery after ablation, the prevalence of moderate/severe post-procedural pain (defined as NRS score ≥ 4) at the recovery room, and the NRS score at arrival recovery. The NRS ranges from zero (no pain) to ten (maximal pain), and was recorded by the nurses in the recovery room. The NRS was measured when the patient arrived and left the recovery room, and sometimes during their stay when needed. Other outcomes included total anesthesia time, total procedure duration time, complications during the ablation (e.g. pneumothorax, perforation, and hematoma), and the need for analgesics during the stay at the recovery room. This was registered when one or more analgesics was used for pain treatment.

When a patient had multiple lesions, the maximal lesion diameter was determined by selecting the largest diameter among these lesions in coronal, sagittal, or transversal views. Also, when a patient had one or more lesions located subcapsular (within 5 mm of the liver capsular) this patient was placed in the subcapsular group, since ablation of subcapsular lesions is expected to result in higher pain intensity compared to deep seeded lesions [13]. The same applied to patients with one or more lesions against a blood vessel located, these were placed in the “against blood vessel” group.

### Statistical Analysis

Patients’ characteristics are described using standard descriptive statistics. Continuous variables were checked on normality using histograms and compared between groups using the independent-samples t-test or the Mann–Whitney U test, depending on the distribution. Categorical and binary variables were tested using Pearson’s chi-squared or Fisher’s exact test when appropriate.

The outcomes were analyzed using multiple ordinal/linear/logistic regression as appropriate. The measures of association are expressed as beta-estimates (ß) or odds ratios (OR) with their 95% confidence interval (CI). We checked for normality of distribution of the residuals using Q–Q plots. When no normality was seen the outcomes were transformed using the natural logarithm. The regression coefficient was than exponentiated to receive ratios. To calculate relative percentages, the following formula was used:

$$\left( {{\text{exponentiate}}\left( {{\text{coefficient}}} \right) - 1} \right)*100\%$$

All regression models were adjusted for age, ASA-score, BMI, tumor type (primary or secondary tumor), maximum diameter of lesion, chronic pain in patients’ history, and history of psychological disorder (depression, sleep disorder or anxiety). The tumor type, anesthesia technic, and maximal lesion diameter were assessed for its role as potential confounder. All analyses were performed using R (version 4.1.2). Alpha was set at 0.05.

### Missing Data

The crude data were used for the descriptive analyses. For the regression analyses, multiple imputation by chained equations (MICE), using the *mice* package in R, were used to impute missing data. The number of imputations was set to 50. Variables used as predictors are shown in Supplemental file 1. Missing data in baseline characteristics are shown in Table 1. In 11 cases (6.0%), the maximal NRS was missing.

**Table 1:** Patients baseline characteristics

	Men (*n* = 123)	Women (*n* = 60)	*P*-value	Missing (%)
Age in years—mean (SD)	69 (9.5)	65 (9.7)	0.003	0.0
BMI in kg/m^2^—mean (SD)	29 (5.3)	26 (4.1)	< 0.001	0.0
Pre chemotherapy—n (%)	31 (67)	26 (72)	0.818	55
ASA-score—n (%)			0.018	0.0
2	33 (27)	28 (47)		
3	86 (70)	29 (48)		
4	4 (3.3)	3 (5.0)		
Chronic pain—n (%)	17 (14)	14 (23)	0.161	0.0
History of psychological disorder—n (%)	7 (5.7)	13 (22)	0.003	0.0
Anesthesia—n (%)			0.362	0.0
Sedation	96 (78)	51 (85)		
General anesthesia	27 (22)	9 (15)		
Tumor type—n (%)			0.005	0.0
Primary	76 (62)	23 (38)		
Cirrhotic livers	65 (86)	15 (65)		
Secondary	47 (38)	37 (62)		
Total lesions—n (%)			0.842	0.0
1	83 (68)	43 (72)		
2	30 (24)	13 (22)		
3	10 (8.1)	4 (6.7)		
Recurrence lesions—n (%)	26 (21)	10 (17)	0.606	
Location lesions—n (%)			0.834	0.0
Deep located	63 (51)	29 (48)		
Subcapsular/exophytic	60 (49)	31 (52)		
Maximal diameter of lesion(s)—mean (SD)	20.9 (7.4)	17.9 (6.7)	0.007	0.0
Vessel involvement			0.256	0.01
> 5 mm distance	67 (55)	40 (67)		
< 5 mm distance	27 (22)	8 (13)		
Against blood vessel	28 (23)	12 (20)		
Imaging used—n (%)*			0.737	0.01
Ultrasound	68 (55)	31 (53)		
CT	29 (24)	17 (29)		
Combined	26 (21)	11 (19)		

*BMI*, body mass index; *ASA*, America Society of Anesthesiologists; *CT*, computed tomography

^*^used imaging was missing in one patient

## Results

### Patient Characteristics

**Fig. 1:**
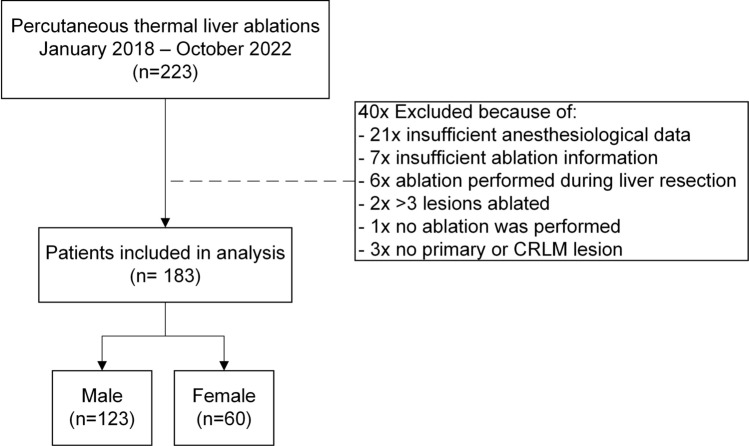
Flowchart of included men and women CRLM: colorectal liver metastasis

After applying in- and exclusion criteria, a total of 183 out of the initial 225 patients were included, of whom 67% were men (Fig. 1). At baseline, men were, on average, older, had higher BMI levels, higher ASA-scores, suffered more often from primary liver tumors, and had larger mean maximal lesion diameters (Table 1). Based on univariable analyses, chronic pain in patients’ history (*P* < 0.05) and history of psychological disorder (*P* < 0.001) were significantly associated with the maximal NRS, and with the abovementioned baseline differences included in the multivariable analyses. Table S1 shows the distribution of men and women among the anesthesia care providers treating anesthesiologists, without significant differences (*P* = 0.70).

### NRS Outcomes

At the recovery, the mean maximal NRS score was significantly higher in women patients (3.88, SD:v2.67) compared to men (2.73, SD:2.64), but not after adjustments; aß:0.75 (95%CI:− 0.13–1.64) (Tables 2, 3). There was no significant interaction between sex and the tumor type, between sex and anesthesia technique, and between sex and maximal lesion diameter on the primary outcome (*P* = 0.82, *P* = 0.72, and *P* = 0.53, respectively). Women had higher rates of post-procedural pain compared to men (59% versus 35%, *P* < 0.01), regression analysis showed a 2.50 higher odds of developing post-procedural pain in women (aOR:2.50, 95%CI:1.16–5.39). Although women patients had on average higher NRS scores at recovery arrival (2.59 versus 1.72), regression analyses showed no substantial difference after adjusting, aß:0.37 (95%CI:−0.48–1.22).

**Table 2:** NRS and procedure related outcomes

	Men (n = 123)	Women (n = 60)	P-value
Maximal NRS score at the recovery—mean (SD)	2.73 (2.64)	3.88 (2.67)	0.008
NRS score at arrival recovery—mean (SD)	1.72 (2.42)	2.59 (2.78)	0.046
Postprocedural pain (NRS 4–10)—n (%)	39 (35)	35 (59)	0.003
Analgesics needed at recovery—n (%)	77 (63)	46 (77)	0.083
Duration of stay recovery—mean (SD)	111 (58)	108 (45)	0.818
Duration of anesthesia—mean (SD)	98 (35)	96 (38)	0.741
Duration procedure—mean (SD)	69 (34)	69 (33)	0.919
Maximal ablation duration*—mean (SD)	570 (148)	555 (146)	0.511
Complications—n (%)	19 (15)	11 (18)	0.778

*NRS*, numerical rating scale

^*^The maximal ablation duration indicates the maximal time of ablation of one lesion during a session without interruption

**Table 3:** Regression analyses on NRS and procedural outcomes

Men as comparator	EE	Unadjusted	Adjusted*
NRS maximal at recovery	ß	1.10 (0.26–1.93)	0.75 (− 0.13–1.64)
NRS 0–3/4–10	OR	2.61 (1.36–5.04)	2.50 (1.16–5.39)
NRS at arrival recovery	ß	0.90 (0.10–1.69)	0.37 (− 0.48–1.22)
Length of stay recovery	%	− 3.33 (− 16–11)	− 1.19 (− 15–15)
Analgesics needed at recovery	OR	1.96 (0.97–3.98)	2.43 (1.07–5.48)
Duration anesthesia	%	− 3.59 (− 14–8.0)	− 1.79 (− 13–11)
Duration procedure	%	0.01 (− 14–17)	− 0.33 (− 16–18)
Ablation duration	ß	− 15 (− 61–31)	18 (− 32–69)
Complications	OR	1.23 (0.54–2.80)	0.89 (0.35–2.72)

*NRS,* numerical rating scale; *EE,* effect estimates

^*^adjusted for age, ASA-score, BMI, primary/secondary tumor, maximum diameter of lesion, chronic pain in patients’ history, history of psychological disorder

### Procedure Outcomes

Women patients more often needed analgesia during the post-procedural stay at the recovery (77% versus 63%; aOR: 2.43, 95%CI:1.07–5.48). Table S2 presents all given analgesia (with their dosage when available) during the procedure, only metamizole was used more in women compared to men patients (8.3% versus 0.0%, P < 0.01). Regression analyses with transformed data showed nonsignificant results regarding the procedure duration, the length of stay at the recovery, and the anesthesia duration in women compared to men (Table 3). During thermal ablation, the complication rates were similar between men and women (15% versus 18%, *P* = 0.78). Table S3 overviews the outcomes in patients with a complication during the procedure. Especially the NRS score at arrival recovery is higher in women with a complication compared to women without a complication (3.91 versus 2.59).

## Discussion

This study investigated pain experiences after thermal liver ablation between men and women. Women experienced higher maximum pain scores, had more often moderate to severe pain, and needed more often analgesics in the recovery room for pain treatment.

On the NRS, women exhibited a higher average maximal pain intensity (3.88 versus 2.73) and a higher prevalence of post-ablation pain (NRS 4–10) compared to men. It has been considered that a minimum change of 1.39 in NRS score is clinically relevant, meaning that our difference in maximal pain intensity might not reach this threshold [14]. However, an NRS ≥ 4 may be more clinically relevant because these patients potentially need pain treatment [15]. This substantiates our results, since women suffered more from postablation pain and needed analgesia more often post-procedurally. Altogether, this data suggests that sex is a relevant post-ablation pain predictor, urging anesthesia care providers to consider it during the procedure to reduce post-ablation pain.

A prior study found higher pain intensities immediately after thermal ablation in patients treated for HCC within two centimeters of the parietal peritoneum [13]. The parietal peritoneum was defined as the abdominal wall at a level below the main portal bifurcation. Above this level, it was defined as the diaphragm, which was not associated with higher pain experiences (*P* = 0.90) [13]. In our study, women were equally exposed to subcapsular tumors compared to men (52% versus 49%; Table 1), limiting the potential influence of tumor location on the pain intensities. Besides, univariable analysis showed no association between the location of the tumor and post-procedural pain.

In general, a larger ablation zone is desired in patients with CRLM, requiring a more aggressive thermal ablation [2, 16]. This may influence the pain intensity in patients, since larger ablation volumes are related to post-ablation pain [12]. In our study, women suffered more from metastases compared to men (38% versus 62%), potentially explaining the higher pain intensity levels. However, there was no significant interaction term between the maximal NRS score and the tumor type, suggesting a limited effect of tumor type on pain intensity. Additionally, we adjusted for tumor type in our regression model to minimize this potential effect.

In surgical settings, pre-operative pain predictors are well-studied. As mentioned earlier, various factors, such as female sex, smoking, higher BMI, younger age, anxiety, history of pre-operative pain, and expectations of post-operative pain, are known to have a negative relation with post-operation pain control [10, 11, and 17]. In our study, besides female sex, history of pre-operative pain, and psychological disorder were potential pain predictors. The reason for this might be the limited influence of such factors in minimally invasive procedures compared to liver surgery on post-procedural pain. During thermal liver ablation, three moments are known to cause pain: skin puncture, liver capsule puncture, and thermal energy transfer [18]. It is conceivable that the number of pain events is significantly higher in liver surgery. This might result in the release of higher levels of inflammatory mediators in liver surgery compared to minimal invasive thermal liver ablation. Such mediators activate peripheral nociceptors and cause peripheral and central sensitization, leading to acute and post-operative pain [19, 20]. When lower amounts of mediators are released, fewer factors may influence this sensitization, thus causing post-procedural pain.

Multiple studies have compared different sedation techniques and analgesics during thermal liver ablations. One study showed lower post-ablation pain in patients treated with intravenous oxycodone versus fentanyl [21]. A Chinese study compared remifentanil plus propofol versus oxycodone and found lower visual analog scale (VAS) scores in patients treated with remifentanil and propofol, whereas another study showed lower VAS scores in patients treated with propofol sedation or general anesthesia versus midazolam sedation [22, 23]. These results suggest that different anesthesia techniques and analgesics result in different post-ablation pain experiences, rendering the comparison of experienced pain very difficult. In our study propofol sedation in combination with a short acting opioid (in most cases remifentanil) was used in the majority of the patients. No statistically significant differences were observed in the use of analgesics between women and men except for metamizole, see Table S2. Metamizole was used more in women compared to men (8.3% versus 0.0%, *P* < 0.01). The higher use of metamizole during thermal ablation may explain the higher post-procedural pain scores in women.

The reasons why women and men experience different levels of pain are still an ongoing research topic. Sex hormones, genotype, and endogenous opioid function may all play a potential role in these differences in pain experiences [24]. A meta-analysis reported sex-specific differences in morphine-induced pain treatment, with women responding better. However, most studies included in this meta-analysis have focused on opioid consumption rather than pain relief [25]. Until more aspects have been thoroughly investigated, anesthesia care providers need to consider post-procedural pain differences in women undergoing thermal liver ablation. They should consider higher dosages of analgesics, possible morphine acting opioids, to reduce post-procedural excessive pain.

Some limitations must be mentioned. First, this single-center study used retrospectively collected data with a subjective pain measurement. Secondly, anesthesia care providers were free to choose different analgesics during the thermal ablation procedure, potentially causing inconsistent sedation levels. As shown above, comparable medications, dosage, and sedation techniques were used in both groups, and the distribution of men and women was equal among treating anesthesia care providers minimalizing this potential bias. Thirdly, some bias may occur, since two different ablation systems were used, the influence of these different ablation techniques could not be taken into account. However, Table S4 shows no difference in the proportion of men or women treated with each ablation system, therefore influence is probably limited.

## Conclusion

This retrospective single-center study shows higher post-procedural pain rates after thermal liver ablation in women, resulting in higher analgesics use at the recovery room. To improve clinical outcome and reduce post-procedural pain, the results suggest considering higher dosage of analgesics during thermal liver ablation in women.

## Supplementary Information

Below is the link to the electronic supplementary material.

Supplementary file1 (PDF 87 KB)
