# Supplementary material for: Sex-Differences in Post-Procedural Pain Experiences After Thermal Liver Ablations for Liver Tumors: A Retrospective Study
Source: Cardiovasc Intervent Radiol. 2024 Sep 4;47(11):1485–92. doi: 10.1007/s00270-024-03851-5 (PMC11541288; doi:10.1007/s00270-024-03851-5)
Supplement: Supplementary file 1 — Supplementary file1 (PDF 87 KB) [file 270_2024_3851_MOESM1_ESM.pdf]

<u>Supplemental</u>

Supplemental S1. Variables used as predictors during multiple imputations

Sex, age, BMI, currently smoking, ASA-classification, history of diabetes mellitus, history of cardiovascular disease, history of hypertension, history of chronic pain, use of neo-adjuvant chemotherapy, total lesions, ultrasound or CT guided, anesthesia duration, procedure duration, duration stay at the recovery, NRS maximal at recovery, maximal time of ablation, lesion location, vessel involvement, primary or secondary tumor, sedation or narcosis, recurrence lesion, more or less than one lesion, NRS at arrival recovery, Propofol use during anesthesia, Remifentanil use during anesthesia, Dipidolor use during anesthesia, Paracetamol use during anesthesia, use of analgetic at the recovery.

Table S1. Overview of anesthesia care providers treating male and female patients.

	Male (n=123)*	Female (n=60)*	P-value
**anesthesia care providers – n (%)**			0.702
1	20 (18)	10 (18)	
2	18 (17)	6 (11)	
3	18 (17)	15 (27)	
4	11 (10)	3 (5.4)	
5	28 (26)	15 (27)	
6	4 (3.7)	2 (3.6)	
7	10 (9.2)	5 (8.9)	

*: In fourteen male and in four female patients the anesthesiologist was unknown.

Table S2. Administered analgesics during thermal ablation

	Male (n=123)	Female (n=60)	P-value
**Analgesic – n (%)**			
Propofol	112 (91)	58 (97)	0.280
Midazolam	4 (3.3)	2 (3.3)	1
Remifentanil	104 (85)	51 (81)	1
Alfentanil	5 (4.1)	6 (10)	0.210
Paracetamol	95 (77)	48 (80)	0.815
Diclofenac	15 (12)	4 (6.7)	0.372
Metamizole	0 (0)	5 (8.3)	**0.006**
Esketamine	24 (20)	16 (27)	0.363
Total mg – median (IQR)	12 (9.8-21)	10 (10-16)	0.485
Mg/kg – median (IQR)	0.145 (0.098-0.238)	0.150 (0.115-0.223)	0.781
Dipidolor	73 (59)	42 (70)	0.216
Total mg – median (IQR)	7.0 (4.0-9.0)	6.0 (4.0-8.0)	0.364
MME – median (IQR)	14 (8.0-18)	12 (8.0-16)	0.364
Morphine	29 (24)	11 (18)	0.538
Total mg – median (IQR)	6.0 (4.0-10)	5.0 (4.0-7.0)	0.214
MME – median (IQR)	18 (12-30)	15 (12-21)	0.214
Clonidine	7 (5.7)	3 (5.0)	1
Total mg – median (IQR)	75 (60-75)	75 68-75()	1
Sufenta	5 (4.1)	2 (3.3)	1

Total mg – median (IQR)	0.030 (0.020-0.030)	0.023 (0.021-0.024)	0.280
MME – median (IQR)	90.0	67.5	0.280

MME: morphine milligram equivalent.

Table S3. NRS and procedure related outcomes in patients with a procedural complication.

	Men (n=19)	Women (n=11)
Maximal NRS score at the recovery – mean (SD)	3.06 (3.15)	4.09 (2.59)
NRS score at arrival recovery – mean (SD)	1.83 (2.83)	3.91 (2.74)
Post-procedural pain (NRS 4-10) – n (%)	6 (33)	7 (64)
Analgesics needed at recovery – n (%)	13 (68)	10 (91)
Duration of stay recovery – mean (SD)	151 (73)	131 (60)
Duration of anesthesia – mean (SD)	102 (33)	115 (52)
Duration procedure – mean (SD)	74 (35)	87 (44)
Maximal ablation duration – mean (SD)	526 (163)	611 (110)

NRS: numerical rating scale; EE: effect estimates.

Table S4. Used thermal liver ablation system

	Male (n=123)	Female (n=60)	P-value
**Liver ablation system**			0.508
HS Amica (Hospital Service)	97 (80)	44 (75)	
Neuwave (Johnson & Johnson)	24 (20)	15 (25)	
